# Use of Deep Learning to Predict Final Ischemic Stroke Lesions From Initial Magnetic Resonance Imaging

**DOI:** 10.1001/jamanetworkopen.2020.0772

**Published:** 2020-03-12

**Authors:** Yannan Yu, Yuan Xie, Thoralf Thamm, Enhao Gong, Jiahong Ouyang, Charles Huang, Soren Christensen, Michael P. Marks, Maarten G. Lansberg, Gregory W. Albers, Greg Zaharchuk

**Affiliations:** 1 Department of Radiology, Stanford University, Stanford, California; 2 Center for Stroke Research Berlin, Charité–Universitätsmedizin Berlin, Berlin, Germany; 3 Department of Electrical Engineering, Stanford University, Stanford, California; 4 Department of Neurology, Stanford University, Stanford, California

## Abstract

**Importance:**

Predicting infarct size and location is important for decision-making and prognosis in patients with acute stroke.

**Objectives:**

To determine whether a deep learning model can predict final infarct lesions using magnetic resonance images (MRIs) acquired at initial presentation (baseline) and to compare the model with current clinical prediction methods.

**Design, Setting, and Participants:**

In this multicenter prognostic study, a specific type of neural network for image segmentation (U-net) was trained, validated, and tested using patients from the Imaging Collaterals in Acute Stroke (iCAS) study from April 14, 2014, to April 15, 2018, and the Diffusion Weighted Imaging Evaluation for Understanding Stroke Evolution Study–2 (DEFUSE-2) study from July 14, 2008, to September 17, 2011 (reported in October 2012). Patients underwent baseline perfusion-weighted and diffusion-weighted imaging and MRI at 3 to 7 days after baseline. Patients were grouped into unknown, minimal, partial, and major reperfusion status based on 24-hour imaging results. Baseline images acquired at presentation were inputs, and the final true infarct lesion at 3 to 7 days was considered the ground truth for the model. The model calculated the probability of infarction for every voxel, which can be thresholded to produce a prediction. Data were analyzed from July 1, 2018, to March 7, 2019.

**Main Outcomes and Measures:**

Area under the curve, Dice score coefficient (DSC) (a metric from 0-1 indicating the extent of overlap between the prediction and the ground truth; a DSC of ≥0.5 represents significant overlap), and volume error. Current clinical methods were compared with model performance in subgroups of patients with minimal or major reperfusion.

**Results:**

Among the 182 patients included in the model (97 women [53.3%]; mean [SD] age, 65 [16] years), the deep learning model achieved a median area under the curve of 0.92 (interquartile range [IQR], 0.87-0.96), DSC of 0.53 (IQR, 0.31-0.68), and volume error of 9 (IQR, −14 to 29) mL. In subgroups with minimal (DSC, 0.58 [IQR, 0.31-0.67] vs 0.55 [IQR, 0.40-0.65]; *P* = .37) or major (DSC, 0.48 [IQR, 0.29-0.65] vs 0.45 [IQR, 0.15-0.54]; *P* = .002) reperfusion for which comparison with existing clinical methods was possible, the deep learning model had comparable or better performance.

**Conclusions and Relevance:**

The deep learning model appears to have successfully predicted infarct lesions from baseline imaging without reperfusion information and achieved comparable performance to existing clinical methods. Predicting the subacute infarct lesion may help clinicians prepare for decompression treatment and aid in patient selection for neuroprotective clinical trials.

## Key Points

**Question:**

Can final infarct lesions be predicted using a deep learning model from baseline imaging in patients with acute ischemic stroke?

**Findings:**

In this multicenter prognostic study of 182 patients with acute ischemic stroke, a deep learning model trained with a collection of acute and follow-up images was used to predict the size and location of infarct lesions at 3 to 7 days after baseline without knowledge of subsequent reperfusion status. In patients with minimal and major reperfusion, the model showed comparable performance to current clinical state-of-the-art methods.

**Meaning:**

This study found that a deep learning model may provide individualized infarct lesion prediction for patients with acute ischemic stroke before intervention.

## Teaser

This prognostic study assesses whether a deep learning model can predict final infarct lesions using magnetic resonance images acquired at initial presentation and compares the model with current clinical prediction methods among patients with acute ischemic stroke.

## Introduction

Stroke is a leading cause of mortality and disability worldwide, with a global lifetime risk of approximately 25%.^1^ Reperfusion therapies, such as intravenous tissue plasminogen activator and thrombectomy, are the only effective treatments to reverse the ischemic changes. Time was initially considered to be the single key factor in acute stroke treatment triaging.^2,3^ More recently, clinical trials, such as the Diffusion and Perfusion Imaging Evaluation for Understanding Stroke Evolution Study (DEFUSE)^4,5,6^ and Extending the Time for Thrombolysis in Emergency Neurological Deficits (EXTEND)^7^ trials, have shown the value of identifying viable tissue based on imaging criteria. Therefore, understanding how baseline imaging can indicate tissue fate is important to appropriate triage of patients with stroke.

At present, patient selection for endovascular therapy is commonly performed using the diffusion-perfusion mismatch paradigm on the imaging acquired at initial presentation (baseline imaging). This process defines 2 classes of tissue: the ischemic core, which is presumed to be irreversibly damaged, visualized on diffusion-weighted imaging (DWI) and quantified using the apparent diffusion coefficient (ADC); and the penumbra, which is the region at risk of infarction in the absence of rapid reperfusion, visualized on perfusion-weighted imaging (PWI) and quantified using the perfusion parameter time to maximum of the residue function (Tmax). Clinical trials using simple thresholded values of these imaging parameters have identified thresholds for ADC (<620 × 10^−6^ mm^2^/s) and Tmax (>6 seconds), and these have been incorporated into clinically available software packages.^8^ Despite the simplicity of single-valued thresholds to predict tissue outcome, such approaches can fail to capture the complexity of acute ischemic stroke. Although advances have been made to automate the segmentations produced by these software programs, they often still require human interpretation and manual editing to remove nonphysiological signals, such as periventricular and contralateral lesions.

Machine learning is a class of computer algorithms that can automatically learn from data without explicit programming. Some initial studies have shown that machine learning can be used to predict stroke lesions.^9,10,11,12,13^ Convolutional neural networks are a subtype of machine learning that does not require humans to define relevant features but instead learns them from data in a training set. Most convolutional neural networks use many hidden layers (hence the term *deep learning*) for nonlinear processing and extraction of important features.^14^ Deep learning has shown impressive results on a wide range of computer vision tasks, and these are beginning to be applied successfully to medical imaging data.^14,15,16,17,18^ One type of deep convolutional neural network architecture known as a U-net has shown much promise for segmentation tasks in medical imaging, owing to its high computational efficiency, sensitivity, and accuracy for image segmentation tasks.^19,20^

In this study, we used a U-net to predict final infarct lesions in patients with acute ischemic stroke, with the initial magnetic resonance images (MRIs) serving as inputs to the model. Although the premise of the diffusion-perfusion mismatch is all-or-none reperfusion, such patients only account for a small subgroup of all patients who undergo reperfusion therapy. This severely limits the number of cases available for training. In this study, we trained a model with all available stroke cases and reported its performance regardless of reperfusion status. We view this process as a first step to produce generalized and individualized prediction for patients with acute ischemic stroke and an important interim step to move toward models that will also incorporate clinical information.

## Methods

### Patient Population

Patients with acute ischemic stroke were enrolled from the Imaging Collaterals in Acute Stroke (iCAS) study from April 14, 2014, to April 15, 2018,^21^ and the Diffusion Weighted Imaging Evaluation for Understanding Stroke Evolution Study–2 (DEFUSE-2) study from July 14, 2008, to September 17, 2011 (results reported in October 2012).^22^ The iCAS study is an ongoing multicenter observational study enrolling patients with clinical symptoms of acute ischemic stroke (≤24 hours from the last well appearance) attributable to the anterior circulation who were considered for endovascular treatment. Detailed inclusion and exclusion criteria were reported previously.^23,24^ The DEFUSE-2 trial enrolled patients with acute ischemic stroke within 12 hours of symptom onset and performed endovascular treatment. The protocol of DEFUSE-2 has been reported in previous literature.^5^ The iCAS and DEFUSE-2 studies, as well as the current study, have been approved by the institutional review boards of the participating institutions, and written informed consent was obtained from all patients. This report followed the Standards for Reporting of Diagnostic Accuracy (STARD) reporting guideline.

**Figure 1.**
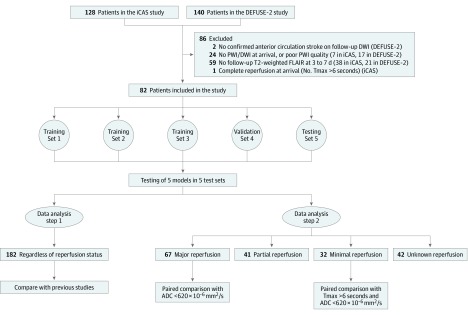
Flow Diagram and Data Analysis Methods During 5-fold cross-validation, patients were randomly divided into 5 sets. In each fold, the 5 sets were split by a ratio of 3:1:1, with 3 sets used for training, 1 for validation, and 1 for testing. No test cases were part of the training or validation sets for any of the 5 folds in the cross-validation. ADC indicates apparent diffusion coefficient; DEFUSE-2, Diffusion Weighted Imaging Evaluation for Understanding Stroke Evolution Study–2; DWI, diffusion-weighted imaging; FLAIR, fluid-attenuated inversion recovery; iCAS, Imaging Collaterals in Acute Stroke; PWI, perfusion-weighted imaging; and Tmax, time to maximum of the residue function.

In this study, we excluded patients if they had (1) no confirmed anterior circulation stroke on follow-up DWI, (2) no PWI or DWI at arrival or PWI of poor quality, (3) no follow-up T2-weighted fluid-attenuated inversion recovery images within 3 to 7 days after stroke onset, or (4) complete reperfusion on baseline PWI (no lesion with Tmax >6 seconds). More details are in Figure 1.

### Imaging Protocol

Images were acquired at 1.5-T or 3-T. At presentation and before reperfusion therapy, all enrolled patients underwent baseline MRI, including DWI (with standard [b = 1000 s/mm^2^] diffusion weighting) and PWI using gadolinium-based contrast agents according to each site’s standard protocol. Clinically available postprocessing software (RAPID, version 4.7.1.2 [iSchemaView]) was used to reconstruct perfusion parameter maps, including Tmax, mean transit time, cerebral blood volume, and cerebral blood flow. This software also generated ADC segmentation with a threshold of less than 620 × 10^−6^ mm^2^/s and Tmax segmentation with a threshold of more than 6 seconds. Most patients underwent a follow-up PWI study within 24 hours, which was used to classify patient perfusion status as minimal, partial, or major, as described below. A T2-weighted fluid-attenuated inversion recovery image was obtained 3 to 7 days after stroke onset to determine the final infarct lesion.

### Imaging Analysis

Investigators at a core laboratory reviewed all studies. The final infarct lesions, which were used as ground truth in this study, were segmented on the T2-weighted fluid-attenuated inversion recovery images by a neuroradiologist (G.Z.) blinded to all clinical information.

Patients were classified into reperfusion categories based on the 24-hour PWI study using the following calculation for reperfusion rate:

reperfusion rate = 100% × (1 − [Tmax at 24 hours >6 seconds/Tmax at baseline > 6 seconds])

Patients with a reperfusion rate of no greater than 20% were classified as having minimal reperfusion, and patients with a reperfusion rate of 80% or greater were classified as having major reperfusion.^25,26^ Otherwise, they were classified as having partial reperfusion (if 24-hour PWI images were available) or unknown reperfusion (if not).

### Imaging Preprocessing

All images were coregistered and normalized to Montreal Neurological Institute template space using SPM12 software (Statistical Parametric Mapping, The Wellcome Trust Centre for Neuroimaging). To compare the model performance in patients with minimal and major reperfusion with the current clinical standard of care, the Tmax and ADC segmentations from RAPID software were used. Tissue with impaired diffusion (ADC <620 × 10^−6^ mm^2^/s) was used to predict the final infarct lesion in patients with major reperfusion.^27^ For patients with minimal reperfusion, where the lesion typically grows to the size of the initial perfusion lesion, the combination of the tissue with impaired diffusion and perfusion (Tmax >6 seconds) and ADC of less than 620 × 10^−6^ mm^2^/s was used for final infarct prediction.

For input of the deep learning model, DWI, ADC, Tmax, mean transit time, cerebral blood flow, and cerebral blood volume were normalized by their means. To preserve important information from the absolute value of Tmax and ADC, 2 binary masks were created separately for Tmax of greater than 6 seconds and ADC of less than 620 × 10^−6^ mm^2^/s using simple thresholding.

### Neural Network

An attention-gated U-Net architecture was used in this study (see neural network details in eMethods and eFigure 1 in the Supplement).^11,19^ We combined the traditional U-Net architecture with attention gates^20^ to focus on target structures. A 2.5-dimensional model is used, meaning that 5 consecutive sections are used to predict the probabilities of final infarct on the center section. The ground truth was a binary mask of final infarct lesion of the middle section measured on the 3- to 7-day follow-up image. Image mirroring around the midline was used for data augmentation. The model outputs a probability map with voxel values that ranged from 0 to 1. A value close to 1 indicates the voxel is more likely to be inside the infarct lesion, whereas a value close to 0 indicates the voxel is unlikely to be inside the infarct lesion. Five-fold cross-validation was performed.

### Performance Evaluation

The area under the curve (AUC) was calculated for the deep learning models and Tmax and ADC thresholding methods (see performance evaluation in eMethods in the Supplement). The Dice score coefficient (DSC) reflects the amount of overlap between the prediction and the truth in the following equation:

DSC = 2 × true-positive/(2 × true-positive + false-positive + false-negative)

It ranges from 0 to 1, with higher numbers representing more overlap (see eFigure 2 in the Supplement for examples and more information). The DSC is preferred to the AUC in tasks in which positive and negative samples are significantly imbalanced, as for infarcted voxels in typical patients with stroke. The DSC also gives information not just on the predicted size of the lesion but also on its spatial location, which is important for brain imaging studies.

The DSC, positive predictive value (PPV), sensitivity, specificity, and lesion volume error between the prediction and ground truth were calculated for the RAPID Tmax and ADC thresholding methods and the deep learning model with an infarct threshold probability of 0.5. Given that large lesions can bias the lesion volume size predictions without affecting clinical importance, we also analyzed lesion volume predictions in cases with lesions smaller than 100 mL separately.

Two data analysis steps were performed (Figure 1). First, the models were tested on all patients regardless of reperfusion status. Next, the models were tested in major and minimal reperfusion groups to compare with the current clinical threshold-based methods.

### Statistical Analysis

Data were analyzed from July 1, 2018, to March 7, 2019. Statistical analysis was performed using Stata, version 14.0 (StataCorp LLC). Paired-sample Wilcoxon tests were performed to compare the area under the curve, DSC, PPV, sensitivity, specificity, lesion volume error, and absolute lesion volume error between the deep learning and thresholding methods. Concordance correlation coefficient (ρ value) and Bland-Altman plots were used to analyze the lesion volume predictions. Because infarct sizes were not normally distributed, cubic root transformation was performed for the concordance correlation calculation. The correlation was considered excellent for ρ > 0.70, moderate for ρ = 0.50 to 0.70, and low for ρ < 0.50.^28^ All tests were 2-sided, and the significance level was adjusted to *P* < .003 owing to multiple comparisons using Bonferroni correction.

## Results

We reviewed 268 patients in the iCAS and DEFUSE-2 studies and included 182 in our analysis (85 men [46.7%] and 97 women [53.3%]; mean [SD] age, 65 [16] years) (Figure 1). Thirty-two patients with minimal reperfusion, 41 with partial reperfusion, 67 with major reperfusion, and 42 with unknown reperfusion were identified. Their clinical information is summarized in Table 1. Patients with major reperfusion had fewer middle cerebral artery M2 occlusions (4 of 67 [6.6%] vs 8 of 23 [34.8%]), smaller median baseline DWI lesions (19 [interquartile range {IQR}, 9-47] vs 42 [IQR, 16-131] mL), and larger median mismatch ratios (5.2 [IQR, 2.7-12.6] vs 2.6 [IQR, 1.4-4.8]) than patients with minimal reperfusion.

**Table 1.** Clinical Information on All Patients and Subgroups

Characteristic	Reperfusion status^a^				
	All (N = 182)	Minimal (n = 32)	Major (n = 67)	Partial (n = 41)	Unknown (n = 42)
Male	85 (46.7)	19 (59.4)	34 (50.7)	17 (41.5)	15 (35.7)
Age, mean (SD), y	65 (15)	62 (15)	64 (16)	67 (14)	64 (17)
Hypertension	126 (69.2)	26 (81)	41 (61.2)	31 (75.6)	28 (66.7)
Diabetes	44 (24.2)	8 (25.0)	13 (19.4)	12 (29.3)	11 (26.2)
Dyslipidemia	83 (45.6)	18 (56.3)	28 (41.8)	25 (61.0)	12 (28.6)
Atrial fibrillation	58 (31.9)	8 (25.0)	23 (34.3)	16 (39.0)	11 (26.2)
Site of occlusion^b^					
MCA M1	86 (59.3)	10 (43.5)	38 (62.3)	21 (60.0)	17 (63.0)
MCA M2	21 (14.5)	8 (34.8)	4 (6.6)	4 (11.4)	5 (18.5)
MCA M3	4 (2.8)	0	2 (3.3)	2 (5.7)	1 (3.7)
ICA	34 (23.4)	5 (21.7)	17 (27.9)	8 (22.9)	4 (14.8)
Treatment methods					
IV tPA	101 (55.5)	17 (53.1)	36 (53.7)	22 (53.7)	26 (61.9)
Thrombectomy	139 (76.4)	22 (68.8)	60 (89.6)	31 (75.6)	26 (61.9)
No treatment	19 (10.4)	6 (18.8)	2 (3.0)	4 (9.8)	7 (16.7)
Onset to treatment time, median (IQR), h	6.0 (4.6-8.3)	7.1 (4.7-8.4)	6.0 (4.6-9.2)	5.6 (3.8-7.1)	6.6 (4.9-8.8)
Baseline DWI lesion volume, median (IQR), mL	28 (11-60)	42 (16-131)	19 (9-47)	37 (16-83)	30 (14-61)
Baseline Tmax lesion volume, median (IQR), mL	116 (67-168)	105 (56-153)	111 (69-153)	156 (80-210)	123 (82-171)
PWI:DWI mismatch ratio, median (IQR)^c^	3.9 (2.2-9.7)	2.6 (1.4-4.8)	5.2 (2.7-12.6)	3.8 (2.5-7.0)	3.6 (2.1-8.9)
Baseline NIHSS score, median (IQR)^d^	15.0 (10.0-19.0)	13.0 (8.5-21.0)	15.0 (10.0-19.0)	17.0 (12.0-20.0)	14.0 (11.0-19.0)
Symptomatic hemorrhage	13 (7.1)	4 (12.5)	4 (6.0)	2 (4.9)	3 (7.1)
Reperfusion rate, median (IQR), %	74 (29-100)	0 (0-11)	100 (96-100)	56 (43-68)	NA
Final infarct volume, median (IQR), mL	54 (16-117)	86 (35-257)	23 (9-64)	82 (27-163)	52 (21-109)
mRS score at 90 d, median (IQR)^e^	3 (1-4)	3 (1-4)	2 (1-3)	4 (3-5)	3 (1-4)

Abbreviations: DWI, diffusion-weighted imaging; ICA, internal carotid artery; IQR, interquartile range; IV tPA, intravenous tissue plasminogen activator; MCA, middle cerebral artery; mRS, modified Rankin scale; NA, not applicable; NIHSS, National Institutes of Health Stroke Scale; PWI, perfusion-weighted imaging; Tmax, time to maximum of the residue function.

^a^ Unless otherwise indicated, data are expressed as number (percentage) of patients.

^b^ Identified on initial digital subtraction angiography. Only 80% of patients received initial digital subtraction angiography.

^c^ The upper limit of mismatch ratio was set to 20 if a small or no ischemic core lesion presented on baseline.

^d^ Scores range from 0 to 42, with higher scores indicating more severe stroke symptoms.

^e^ Scores range from 0 to 6, with higher scores indicating worse functional outcome.

### Model Performance in All Patients

**Figure 2.**
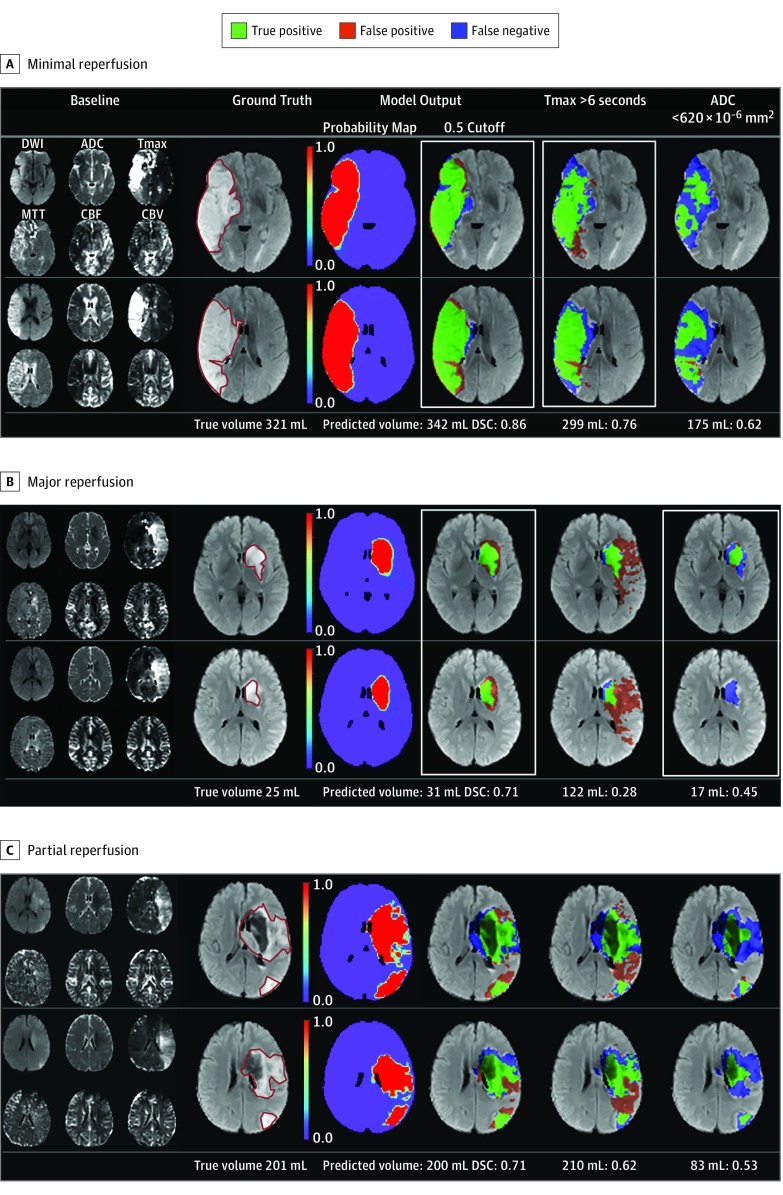
Examples of Predictions From the Model Compared With Thresholding Methods in Typical Cases Two representative sections are shown. Minimal reperfusion indicates a 24-hour reperfusion rate of 0%; major reperfusion, 24-hour reperfusion rate of 100%; and partial reperfusion, 24-hour reperfusion rate of 60%. Baseline images acquired at presentation were inputs, and the final true infarct lesion at 3 to 7 days was considered the ground truth for the model. Infarct lesions at 3 to 7 days are outlined by the red solid line on the T2-weighted fluid-attenuated inversion recovery images. Numbers after predicted volume (mL) indicate Dice score coefficients. CBF indicates cerebral blood flow; CBV, cerebral blood volume; DSC, Dice score coefficient; DWI, diffusion-weighted imaging; MTT, mean transit time; and Tmax transit, time to maximum of the residue function.

The deep learning model had a median area under the curve of 0.92 (IQR, 0.87-0.96). Using a threshold of 0.50, the model had a median DSC overlap of 0.53 (IQR, 0.31-0.68), sensitivity of 0.66 (IQR, 0.38-0.86), specificity of 0.97 (IQR, 0.94-0.99), PPV of 0.53 (IQR, 0.28-0.74), volume error of 9 (IQR, –14 to 29) mL, and absolute volume error of 24 (IQR, 11-50) mL. The volume predicted from the model had excellent correlation with true lesion volume (ρ = 0.74; 95% CI, 0.66-0.80) (eFigure 3 in the Supplement). The lesion volume prediction of the model across all subgroups was more consistently stable than for the clinical thresholding models (ADC or ADC plus Tmax) (eFigure 4 in the Supplement). Representative typical cases are shown in Figure 2 and atypical cases in eFigure 5 in the Supplement.

### Model Performance in Patients With Minimal and Major Reperfusion 

**Figure 3.**
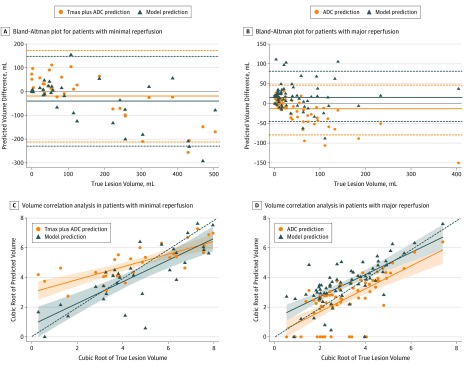
Bland-Altman Plots and Volume Correlation Analyses From the Deep Learning Model and Thresholding Methods in Patients With Minimal and Major Reperfusion A and B, The solid lines represent mean and the dotted lines marking the shaded areas represent upper and lower limit of agreement. C and D, Because the data are not normally distributed in the volume correlation analysis, the cubic root of the volume is plotted for clarity. In part D, apparent diffusion coefficient (ADC) thresholding erroneously predicted a zero lesion size more commonly than the deep learning model (13 vs 2 patients, respectively). Solid lines indicate fitted linear regression line; dashed lines, reference lines when the correlation coefficient equals 1; and shaded area, 95% CI of fitted linear regression from the SE of the prediction.

Performance metrics for the patients with minimal and major reperfusion compared with thresholding methods can be found in Table 2. In patients with minimal reperfusion, we found no difference between predicted and true lesion volume for the proposed model (difference, −3 [IQR, −84 to 18]; *P* = .07) or the Tmax plus ADC segmentation (difference, 9 [IQR, −72 to 55]; *P* = .90). Volume prediction from Tmax plus ADC segmentation (ρ = 0.65; 95% CI, 0.47-0.77) and the model (ρ = 0.76; 95% CI, 0.58-0.87) yielded moderate and excellent agreement, respectively, with true lesion volume. When compared with Tmax plus ADC segmentation, the proposed model had higher PPV and specificity. In 17 patients with a ground truth lesion volume of less than 100 mL, the proposed model had a median volume error of 6 (IQR, −5 to 16) mL, whereas Tmax plus ADC segmentation overestimated the lesion volume by 32 (IQR, 8-61) mL. In 15 patients with lesions greater than 100 mL, the proposed model and Tmax plus ADC segmentation underestimated the lesion volume (−90 [IQR, −200 to 21] mL and −73 [IQR, −169 to 10] mL, respectively) (Figure 3).

**Table 2.** Model Performance and Comparison Between Model and Tmax and ADC Methods

Perfusion Model	Median (IQR)						
	AUC	DSC	PPV	Sensitivity	Specificity	Volume Error, mL	
						Lesion	Absolute Lesion
Minimal (n = 32)	0.90 (0.85 to 0.94)	0.58 (0.31 to 0.67)	0.71 (0.39 to 0.85)	0.54 (0.36 to 0.80)	0.97 (0.95 to 0.99)	−3 (−84 to 18)	39 (13 to 107)
Tmax >6 s plus ADC <620 × 10^−6^ mm^2^/s	0.80 (0.74 to 0.85)	0.55 (0.40 to 0.65)	0.46 (0.33 to 0.81)	0.57 (0.47 to 0.74)	0.94 (0.90 to 0.96)	9 (−72 to 55)	59 (23 to 100)
*P* value	<.001	.37	.002	.43	.002	.04	.61
Major (n = 67)	0.93 (0.89 to 0.96)	0.48 (0.29 to 0.65)	0.41 (0.21 to 0.64)	0.74 (0.50 to 0.80)	0.97 (0.95 to 0.99)	16 (−3 to 29)	19 (10 to 33)
ADC <620 × 10^−6^ mm^2^/s	0.71 (0.59 to 0.78)	0.45 (0.15 to 0.54)	0.44 (0.13 to 0.72)	0.42 (0.19 to 0.59)	0.99 (0.98 to 1)	−8 (−34 to 6)	12 (7 to 34)
*P* value	<.001	.002	.02	<.001	<.001	<.001	.26
Partial (n = 41)	0.90 (0.86 to 0.96)	0.53 (0.36 to 0.68)	0.59 (0.38 to 0.82)	0.61 (0.42 to 0.81)	0.96 (0.92 to 0.98)	9 (−31 to 37)	36 (15 to 66)
Unknown (n = 42)	0.92 (0.86 to 0.96)	0.52 (0.31 to 0.67)	0.54 (0.33 to 0.76)	0.60 (0.39 to 0.86)	0.96 (0.94 to 0.99)	6 (−11 to 32)	26 (10 to 38)

Abbreviations: ADC, apparent diffusion coefficient; AUC, area under the curve; DSC, Dice score coefficient; IQR, interquartile range; PPV, positive predictive value; Tmax, time to maximum of the residue function.

In patients with major reperfusion, the proposed model overestimated lesion volume (difference, 16 [IQR, −3 to 29] mL; *P* < .001), whereas the ADC method underestimated volume (difference, −8 [IQR, −34 to 6] mL; *P* < .001). Volume prediction from ADC (ρ = 0.63; 95% CI, 0.47-0.74) and the model (ρ = 0.67; 95% CI, 0.52-0.78) were similar. When compared with ADC thresholding, the proposed model had higher DSC and sensitivity but lower specificity. In 57 patients with ground truth lesions of less than 100 mL, the proposed model overestimated the lesion size by 16 (IQR, 0-25) mL compared with that of ADC segmentation by −2 (IQR, −17 to 7) mL; in 9 patients with lesions of greater than 100 mL, the model overestimated the lesion by 16 (IQR, −6 to 37) mL, whereas ADC segmentation underestimated by −59 (IQR, −89 to −39) mL (Figure 3).

### Model Performance in Patients With Partial and Unknown Reperfusion

In patients with partial and unknown reperfusion status, the model had moderate to excellent agreement between predicted lesion volume (ρ = 0.69 [95% CI, 0.51-0.82]) and true lesion volume (ρ = 0.75 [95% CI, 0.58-0.86]). Volumetrically, the proposed model did not show a significant difference from the true lesion (proposed volume error, 9 [IQR, −31 to 37] mL; true lesion volume error, 6 [IQR, −11 to 32] mL).

## Discussion

Our study demonstrated that an attention-gated U-net deep learning model trained using only baseline multisequence MRI data could be used to predict 3- to 7-day infarct lesions. The model was trained without including information about reperfusion status, yet it had comparable performance in patients with and without major reperfusion compared with a common clinically used ADC and Tmax thresholding software package. Furthermore, it performed similarly well in patients with partial or unknown reperfusion status, among whom neither of the traditional prediction methods based on the diffusion-perfusion mismatch paradigm apply.

In patients with minimal reperfusion, the proposed model outperformed the clinical thresholding method for PPV and specificity while maintaining comparable DSC and sensitivity. For lesions of less than 100 mL, in which small differences are clinically most relevant, the proposed model predicted volume more accurately than the clinical thresholding method. For those patients with major reperfusion, the proposed model outperformed the clinical thresholding method for DSC and sensitivity. In these patients, the model tended to overestimate the final infarct lesion, whereas the ADC segmentation tended to underestimate the lesion. The clinical ADC thresholding method outperformed the proposed model for specificity, which is expected, because the area of infarct at baseline rarely shows reversibility. For example, only 1 patient in our cohort demonstrated any ADC reversal (patient B in eFigure 5 in the Supplement), which can occur immediately after reperfusion, but which does not generally persist.^29^ The performance of the proposed model is significantly better than that reported in the previous literature, with almost twice the overlap of the predicted and true lesions of these earlier methods^9,30^ (for more detail, see the discussion in eMethods in the Supplement).

Although imaging features at baseline may be associated with successful therapy,^31,32,33^ the effect of treatment and subsequent infarct growth is difficult to predict.^34^ The prediction of the proposed model may act as a “most likely” final infarct for patients on arrival, given the most common treatment decisions and their success rate, which can provide additional information other than mismatch profile for the decision-making. Because the proposed model predicts the infarct lesion at 3 to 7 days, when the size of the lesion is largest owing to acute vasogenic edema, it would be helpful to guide treatment decisions and coordinate clinical resources such as early preparation for decompression surgery and osmotherapy. In addition, as recanalization rates improve and the treatment time window becomes longer, neuroprotective agents become an important next step for acute ischemic stroke research.^35^ Patient selection for future clinical trials of neuroprotective agents based on imaging also becomes relevant. Our proposed model, providing a comprehensive estimation of the subacute stroke lesion that includes area of edema and hemorrhagic transformation, could serve as a marker for the patient selection in such trials. Further studies are warranted to explore the association between model prediction and outcomes such as cerebral herniation and functional outcome.

The state-of-the-art estimations for penumbra and ischemic core are thresholding methods (Tmax >6 seconds and ADC <620 × 10^−6^ mm^2^/s).^6,36,37^ However, these thresholds are derived from linear analysis and have not been validated in large cohorts.^8,38^ Because factors such as collateral status and gray and/or white matter content may result in different susceptibility to ischemia,^39^ the single-valued threshold may be improved by criteria derived from nonlinear analysis. One way to investigate the nonlinear criteria for penumbra and ischemic core is to train 2 models using only patients with minimal reperfusion or major reperfusion. However, this approach is significantly limited by available case numbers because more than 50% of patients have partial reperfusion where the reperfusion rate at 24 hours is intermediate (20%-80%) or the Thrombolysis in Cerebral Infarction score ranges from 2a to 2b.^6,40,41^ Therefore, we believe a more general model that has learned the association between baseline imaging features and final infarct would be valuable as a starting point for fine-tuning models for specific reperfusion subgroups, resulting in an improved clinical tool for selection of patients with stroke.

### Limitations

This study has several limitations. Treatments varied with respect to the use of thrombectomy, and posterior circulation stroke was not included. Model performance may be affected by the lesion sizes in this cohort and thus might differ in cohorts with other lesion size distributions. However, the studies came from many institutions during a long period, and many different scanners were used, so it is likely that the results generalize better than if they all came from a single site. Next, the ground truth was defined at 3 to 7 days, when vasogenic edema peaks. This may have caused the underestimation on the ADC thresholding approach. However, other follow-up time points also have disadvantages: at 24 hours, the lesion is not fully evolved,^36^ and time points later than 3 to 7 days are difficult to acquire as patients leave the hospital. It is well recognized that stroke outcome depends strongly on reperfusion. We ignored this factor to allow us to train on the largest possible data set and set a performance baseline. Clinical decision-making hinges on the difference between the best and worst therapy outcomes, and ideally training separate models for each of these conditions would provide such information. Future studies should to assess this important area, with the current model serving as a starting point for fine-tuning approaches to achieve this important goal.

## Conclusions

An attention-gated U-net model using baseline MRI was able to predict 3- to 7-day infarction in patients with acute stroke without reperfusion information. It showed comparable performance to the state-of-the-art thresholding methods in patients in whom it might be compared and a similar high level of performance in patients with partial or unknown reperfusion status. Further studies are warranted to validate the results in larger and more diverse cohorts and to incorporate important clinical predictors to improve the model performance.

## Notes

Supplement. **eMethods.** Neural Network Details, Performance Evaluation, and Discussion **eFigure 1.** The Block Diagram of The Attention-Gated U-Net Model and the Schematic of the Attention Gate **eFigure 2.** Example of Cases With Low, Medium, and High Dice Score Coefficient **eFigure 3.** The Correlation of Cubic-Rooted Volume Prediction From Model vs True Lesion Volume **eFigure 4.** Comparison Between the Proposed Deep Learning Model, Tmax + ADC, and ADC Lesion Volume Prediction in Patients With Minimal, Partial, Major, and Unknown Reperfusion **eFigure 5.** Examples of Predictions From Model Compared With Thresholding Methods in Atypical Cases **eReferences.**

## References

[1] Feigin VL, Nguyen G, Cercy K, ; GBD 2016 Lifetime Risk of Stroke Collaborators Global, regional, and country-specific lifetime risks of stroke, 1990 and 2016. N Engl J Med. 2018;379(25):-. doi:10.1056/NEJMoa1804492 30575491 PMC6247346

[2] Hacke W, Kaste M, Bluhmki E, ; ECASS Investigators Thrombolysis with alteplase 3 to 4.5 hours after acute ischemic stroke. N Engl J Med. 2008;359(13):1317-1329. doi:10.1056/NEJMoa0804656 18815396

[3] Saver JL, Goyal M, van der Lugt A, ; HERMES Collaborators Time to treatment with endovascular thrombectomy and outcomes from ischemic stroke: a meta-analysis. JAMA. 2016;316(12):1279-1288. doi:10.1001/jama.2016.13647 27673305

[4] Ogata T, Christensen S, Nagakane Y, ; EPITHET and DEFUSE Investigators The effects of alteplase 3 to 6 hours after stroke in the EPITHET-DEFUSE combined dataset: post hoc case-control study. Stroke. 2013;44(1):87-93. doi:10.1161/STROKEAHA.112.668301 23250996

[5] Lansberg MG, Straka M, Kemp S, ; DEFUSE 2 study investigators MRI profile and response to endovascular reperfusion after stroke (DEFUSE 2): a prospective cohort study. Lancet Neurol. 2012;11(10):860-867. doi:10.1016/S1474-4422(12)70203-X 22954705 PMC4074206

[6] Albers GW, Marks MP, Kemp S, ; DEFUSE 3 Investigators Thrombectomy for stroke at 6 to 16 hours with selection by perfusion imaging. N Engl J Med. 2018;378(8):708-718. doi:10.1056/NEJMoa1713973 29364767 PMC6590673

[7] Ma H, Campbell BCV, Parsons MW, ; EXTEND Investigators Thrombolysis guided by perfusion imaging up to 9 hours after onset of stroke. N Engl J Med. 2019;380(19):1795-1803. doi:10.1056/NEJMoa1813046 31067369

[8] Albers GW, Lansberg MG, Kemp S, A multicenter randomized controlled trial of endovascular therapy following imaging evaluation for ischemic stroke (DEFUSE 3). Int J Stroke. 2017;12(8):896-905. doi:10.1177/1747493017701147 28946832 PMC5916787

[9] McKinley R, Häni L, Gralla J, Fully automated stroke tissue estimation using random forest classifiers (FASTER). J Cereb Blood Flow Metab. 2017;37(8):2728-2741. doi:10.1177/0271678X16674221 27798267 PMC5536784

[10] Nielsen A, Hansen MB, Tietze A, Mouridsen K Prediction of tissue outcome and assessment of treatment effect in acute ischemic stroke using deep learning. Stroke. 2018;49(6):1394-1401. doi:10.1161/STROKEAHA.117.019740 29720437

[11] Pinto A, Mckinley R, Alves V, Wiest R, Silva CA, Reyes M Stroke lesion outcome prediction based on MRI imaging combined with clinical information. Front Neurol. 2018;9:1060. doi:10.3389/fneur.2018.01060 30568631 PMC6290552

[12] Stier N, Vincent N, Liebeskind D, Scalzo F Deep learning of tissue fate features in acute ischemic stroke. Proceedings (IEEE Int Conf Bioinformatics Biomed). 2015;2015:1316-1321. doi:10.1109/BIBM.2015.7359869 28919983 PMC5597003

[13] Winder AJ, Siemonson S, Flottman F, Fiehler J, Forkert ND Comparison of classification methods for voxel-based prediction of acute ischemic stroke outcome following intra-arterial intervention [published online March 3, 2017]. Proc SPIE. doi:10.1117/12.2254118

[14] Chilamkurthy S, Ghosh R, Tanamala S, Deep learning algorithms for detection of critical findings in head CT scans: a retrospective study. Lancet. 2018;392(10162):2388-2396. doi:10.1016/S0140-6736(18)31645-3 30318264

[15] Park A, Chute C, Rajpurkar P, Deep learning-assisted diagnosis of cerebral aneurysms using the HeadXNet model. JAMA Netw Open. 2019;2(6):e195600. doi:10.1001/jamanetworkopen.2019.5600 31173130 PMC6563570

[16] Esteva A, Kuprel B, Novoa RA, Dermatologist-level classification of skin cancer with deep neural networks. Nature. 2017;542(7639):115-118. doi:10.1038/nature21056 28117445 PMC8382232

[17] Rajpurkar P, Irvin J, Zhu K, CheXNet: radiologist-level pneumonia detection on chest x-rays with deep learning. arXiv e-prints. https://ui.adsabs.harvard.edu/abs/2017arXiv171105225R. Published November 2017. Accessed November 01, 2017.

[18] Gulshan V, Peng L, Coram M, Development and validation of a deep learning algorithm for detection of diabetic retinopathy in retinal fundus photographs. JAMA. 2016;316(22):2402-2410. doi:10.1001/jama.2016.17216 27898976

[19] Ronneberger O, Fischer P, Brox T U-Net: convolutional networks for biomedical image segmentation. Submitted May 18, 2015. Accessed July 1, 2018. https://arxiv.org/abs/1505.04597

[20] Oktay O, Schlemper J, Folgoc LL, Attention U-Net: learning where to look for the pancreas. Revised May 20, 2018. Accessed November 20, 2018. https://arxiv.org/abs/1804.03999

[21] Imaging Collaterals in Acute Stroke (iCAS). Clinicaltrials.gov identifier: NCT02225730. Updated September 30, 2019. Accessed July 1, 2018. https://clinicaltrials.gov/ct2/show/NCT02225730

[22] Diffusion Weighted Imaging Evaluation for Understanding Stroke Evolution Study-2 (DEFUSE-2). Clinicaltrials.gov identifier: NCT01349946. Updated April 14, 2016. Accessed July 1, 2018. https://clinicaltrials.gov/ct2/show/NCT01349946

[23] Zaharchuk G, Marks MP, Do HM, Introducing the Imaging the Collaterals in Acute Stroke (iCAS) multicenter MRI trial. Stroke. 2015;46:AWMP16. Accessed February 10, 2020. https://www.ahajournals.org/doi/10.1161/str.46.suppl_1.wmp16

[24] Thamm T, Guo J, Rosenberg J, Contralateral hemispheric cerebral blood flow measured with arterial spin labeling can predict outcome in acute stroke. Stroke. 2019;50(12):3408-3415. doi:10.1161/STROKEAHA.119.026499 31619150 PMC7041883

[25] Bivard A, Levi C, Spratt N, Parsons M Perfusion CT in acute stroke: a comprehensive analysis of infarct and penumbra. Radiology. 2013;267(2):543-550. doi:10.1148/radiol.12120971 23264345

[26] Yu Y, Han Q, Ding X, Defining core and penumbra in ischemic stroke: a voxel- and volume-based analysis of whole brain CT perfusion. Sci Rep. 2016;6:20932. doi:10.1038/srep20932 26860196 PMC4748242

[27] Yu Y, Guo D, Lou M, Liebeskind D, Scalzo F Prediction of hemorrhagic transformation severity in acute stroke from source perfusion MRI. IEEE Trans Biomed Eng. 2018;65(9):2058-2065. doi:10.1109/TBME.2017.2783241 29989941

[28] Mukaka MM Statistics corner: a guide to appropriate use of correlation coefficient in medical research. Malawi Med J. 2012;24(3):69-71. Accessed February 10, 2020. https://www.ncbi.nlm.nih.gov/pmc/articles/PMC3576830/23638278 PMC3576830

[29] Inoue M, Mlynash M, Christensen S, ; DEFUSE 2 Investigators Early diffusion-weighted imaging reversal after endovascular reperfusion is typically transient in patients imaged 3 to 6 hours after onset. Stroke. 2014;45(4):1024-1028. doi:10.1161/STROKEAHA.113.002135 24558095 PMC4396865

[30] Winzeck S, Hakim A, McKinley R, ISLES 2016 and 2017: benchmarking ischemic stroke lesion outcome prediction based on multispectral MRI. Front Neurol. 2018;9:679. doi:10.3389/fneur.2018.00679 30271370 PMC6146088

[31] Bang OY, Saver JL, Kim SJ, Collateral flow predicts response to endovascular therapy for acute ischemic stroke. Stroke. 2011;42(3):693-699. doi:10.1161/STROKEAHA.110.595256 21233472 PMC3051344

[32] Seners P, Delepierre J, Turc G, ; PREDICT-RECANAL Collaborators Thrombus length predicts lack of post-thrombolysis early recanalization in minor stroke with large vessel occlusion. Stroke. 2019;50(3):761-764. doi:10.1161/STROKEAHA.118.023455 30802186

[33] Menon BK, Al-Ajlan FS, Najm M, ; INTERRSeCT Study Investigators Association of clinical, imaging, and thrombus characteristics with recanalization of visible intracranial occlusion in patients with acute ischemic stroke. JAMA. 2018;320(10):1017-1026. doi:10.1001/jama.2018.12498 30208455 PMC6143104

[34] Zhu G, Michel P, Jovin T, Prediction of recanalization in acute stroke patients receiving intravenous and endovascular revascularization therapy. Int J Stroke. 2015;10(1):28-36. doi:10.1111/ijs.12312 24975168

[35] Savitz SI, Baron JC, Yenari MA, Sanossian N, Fisher M Reconsidering neuroprotection in the reperfusion era. Stroke. 2017;48(12):3413-3419. doi:10.1161/STROKEAHA.117.017283 29146878

[36] Purushotham A, Campbell BC, Straka M, Apparent diffusion coefficient threshold for delineation of ischemic core. Int J Stroke. 2015;10(3):348-353. doi:10.1111/ijs.12068 23802548 PMC3786020

[37] Albers GW, Thijs VN, Wechsler L, ; DEFUSE Investigators Magnetic resonance imaging profiles predict clinical response to early reperfusion: the Diffusion and Perfusion Imaging Evaluation for Understanding Stroke Evolution (DEFUSE) study. Ann Neurol. 2006;60(5):508-517. doi:10.1002/ana.20976 17066483

[38] Olivot JM, Mlynash M, Thijs VN, Optimal Tmax threshold for predicting penumbral tissue in acute stroke. Stroke. 2009;40(2):469-475. doi:10.1161/STROKEAHA.108.526954 19109547 PMC2670783

[39] Murphy BD, Fox AJ, Lee DH, White matter thresholds for ischemic penumbra and infarct core in patients with acute stroke: CT perfusion study. Radiology. 2008;247(3):818-825. doi:10.1148/radiol.2473070551 18424687

[40] Campbell BC, Mitchell PJ, Kleinig TJ, ; EXTEND-IA Investigators Endovascular therapy for ischemic stroke with perfusion-imaging selection. N Engl J Med. 2015;372(11):1009-1018. doi:10.1056/NEJMoa1414792 25671797

[41] Berkhemer OA, Fransen PS, Beumer D, ; MR CLEAN Investigators A randomized trial of intraarterial treatment for acute ischemic stroke. N Engl J Med. 2015;372(1):11-20. doi:10.1056/NEJMoa1411587 25517348

