# Error in Figure 1

**DOI:** 10.1001/jamanetworkopen.2020.26464

**Published:** 2020-10-22

In the Original Investigation titled “Use of Deep Learning to Predict Final Ischemic Stroke Lesions From Initial Magnetic Resonance Imaging,”^1^ published online March 12, 2020, in Figure 1, the box for “patients included in the study” incorrectly showed 82 patients; the correct number is 182. This article has been corrected.

## References

[1] Yu Y, Xie Y, Thamm T, Use of deep learning to predict final ischemic stroke lesions from initial magnetic resonance imaging. JAMA Netw Open. 2020;3(3):e200772. doi:10.1001/jamanetworkopen.2020.0772 32163165 PMC7068232

