# Supplementary material for: Use of Deep Learning to Predict Final Ischemic Stroke Lesions From Initial Magnetic Resonance Imaging
Source: JAMA Netw Open. 2020 Mar 12;3(3):e200772. doi: 10.1001/jamanetworkopen.2020.0772 (PMC7068232; doi:10.1001/jamanetworkopen.2020.0772)
Supplement: Supplement. — eMethods. Neural Network Details, Performance Evaluation, and Discussion eFigure 1. The Block Diagram of The Attention-Gated U-Net Model and the Schematic of the Attention Gate eFigure 2. Example of Cases With Low, Medium, and High Dice Score Coefficient eFigure 3. The Correlation of Cubic-Rooted Volume Prediction From Model vs True Lesion Volume eFigure 4. Comparison Between the Proposed Deep Learning Model, Tmax + ADC, and ADC Lesion Volume Prediction in Patients With Minimal, Partial, Major, and Unknown Reperfusion eFigure 5. Examples of Predictions From Model Compared With Thresholding Methods in Atypical Cases eReferences. [file jamanetwopen-3-e200772-s001.pdf]

Supplementary Online Content

Yu Y, Xie Y, Thamm T, et al. Use of deep learning to predict final ischemic stroke lesions from initial magnetic resonance imaging. *JAMA Netw Open*. 2020;3(3):e200772. doi:10.1001/jamanetworkopen.2020.0772

eMethods. Neural Network Details, Performance Evaluation, and Discussion
eFigure 1. The Block Diagram of The Attention-Gated U-Net Model and the Schematic of the Attention Gate
eFigure 2. Example of Cases With Low, Medium, and High Dice Score Coefficient
eFigure 3. The Correlation of Cubic-Rooted Volume Prediction From Model vs True Lesion Volume
eFigure 4. Comparison Between the Proposed Deep Learning Model, Tmax + ADC, and ADC Lesion Volume Prediction in Patients With Minimal, Partial, Major, and Unknown Reperfusion
eFigure 5. Examples of Predictions From Model Compared With Thresholding Methods in Atypical Cases
eReferences.

This supplementary material has been provided by the authors to give readers additional information about their work.

©2020 Yu Y et al. *JAMA Network Open*.

eMethods. Neural Network Details, Performance Evaluation, and Discussion

Neural Network Details

As shown in supplemental figure 1, We combined the traditional U-Net architecture with attention-gates[1] to focus on target structures without additional supervision, which was achieved by combining contextual information from output of previous layers (coarser scales) and symmetric encoding layers.

The model was trained with ADAM optimizer (learning rate 0.0005) using a batch size of 16 and 120 epochs. 50% dropout was implemented for preventing overfitting in both encoding and decoding layers[2]. We used a mixed loss function of weighted binary cross-entropy, mean absolute error (L1 loss), Dice score coefficient (DSC), and volume loss as described below in more detail. Since stroke lesions are only present in relatively a small fraction of all brain voxels, weighting was applied to balance the numbers of positive and negative voxels. The weights for positive and negative voxels were calculated based on the ratio of the positive and negative voxels of each training batch:

$$R_0 = \frac{1}{\frac{N_-}{N_+}+1}$$

$$R_1 = 1 - R_0$$

where N_- and N_+ represent the number of negative and positive voxels per batch, respectively.

$$\text{Weighted binary cross entropy} = -\frac{1}{N}\sum_{i=0}^{N} R_1 y_i log(p_i) + R_0(1-y_i)\, log(1-p_i)$$

$$\text{L1 loss} = \sum_{i=0}^{N} |y_i - p_i|$$

$$\text{Dice score coefficient (DSC)} = \frac{2N_{TP}}{2\,N_{TP} + N_{FP} + N_{FN}}$$

$$\text{Volume loss} = \frac{|\sum_{i=0}^{N} p_i - \sum_{i=0}^{N} y_i|}{N_+}$$

p is the predicted probability. *y* is the ground truth value of that voxel (0 = not infarcted, 1 = infarcted). *N* is the total number of pixels. N_{TP}, N_{FP}, and N_{FN} are the number of true positive, false positive, and false negative voxels, respectively.

The loss function was then expressed as:

Loss = Weighted binary cross entropy + L1 loss + 0.5×(1 – DSC) + 0.5×Volume loss

The weight of 0.5 was given to DSC and volume loss to adjust them to a similar scale of the weighted binary cross entropy and L1 loss.

©2020 Yu Y et al. *JAMA Network Open*.

The implementation was based on Keras (version 2.2.2) with Tensorflow (version 1.10.0) backend. All tests were conducted on a workstation equipped with Quadro GV100 and Tesla V100-PCIE graphical processing units (Nvidia, Santa Clara, CA, USA).

Five-fold cross-validation was performed. Patients were randomly divided into five sets. In each fold, the 5 sets were split by a ratio of 3:1:1, with 3 sets used for training, 1 for validation, and 1 for testing. The best model for each training fold was selected based on the best performance in the validation set. Then the evaluation of model prediction was performed on the test set. No test cases were part of the training or validation sets for any of the 5 folds in the cross-validation; i.e., the results are from 5 separate models trained independently with the training/validation sets for that fold. Each fold took approximately 7 hours to train. A prediction map can be generated in approximately 20 sec for each patient once the model was trained (inference).

Performance Evaluation

Area-under-curve (AUC) was calculated for the deep learning model by varying the output probability threshold for classifying a voxel as infarcted tissue. The AUC methodology influences the results greatly due to the overwhelming number of non-infarcted voxels in stroke patients. We adopted two common AUC calculation methods: to compare with Tmax and ADC segmentation, AUC was calculated for each case within the ipsilateral stroke hemisphere, except in one case, where there were bilateral strokes; To enable comparison with previous studies[3], another formulation (AUC_0) was calculated for the model[4], which considered the prediction based on regions inside and outside the hypoperfused areas. Additionally, in patients with minimal and major reperfusion, we calculated an AUC for the clinical thresholding methods using Tmax and ADC. To calculate AUC in minimal reperfusion patients, the Tmax threshold was varied (4s, 6s, 8s, and 10s) with fixed segmentation of $ADC < 620 \times 10^{-6}$ mm^2/s. To calculate AUC in major reperfusion patients, the ADC threshold was varied using the original ADC map.

Discussion

There have been a few prior studies that have tried to predict subacute stroke lesions from baseline data using machine learning or deep learning approaches[3,5-7]. Nielsen et al. trained a deep CNN model in 158 IV tPA treated patients to predict follow-up FLAIR lesions in a test set of 29 patients, reporting an AUC_0 of 0.88±0.12[3], similar to our model's AUC_0 of 0.89 (IQR 0.83, 0.93). Another study, the Ischemic Stroke Lesion Segmentation (ISLES) 2017 challenge[6,8], centered around predicting 90-day FLAIR lesions based on acute imaging. The top performing team achieved a DSC of 0.33, reflecting the difficulty of the task. Using a non-neural network approach, McKinley et al.[5] trained two random forest classifiers on 15 cases with complete recanalization (TICI 3) and 10 cases with permanent occlusion (TICI 0). They reported a DSC of 0.32±0.23 in cases with TICI grade of 1 and 2a and 0.34±0.22 in cases with TICI grade 2b and 3. Compared to these studies, our model had almost 2-fold higher DSC (0.53), likely related to the much larger number of patients available for training.

eFigure 1. The Block Diagram of The Attention-Gated U-Net Model and the Schematic of the Attention Gate

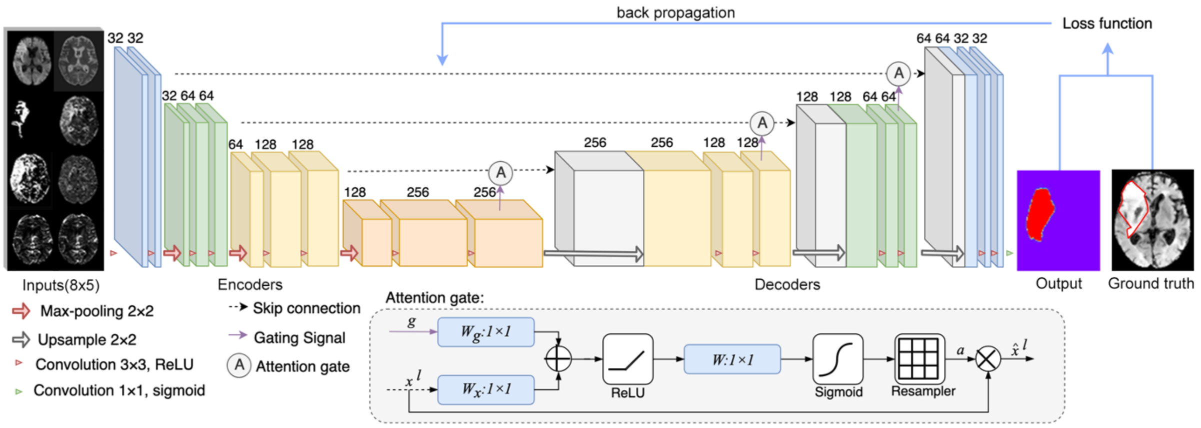

Input images included 5 consecutive slices of diffusion-weighted imaging (b=1000), apparent diffusion coefficient and its thresholded mask with a threshold of less than 620×10^{-6} mm^2/s, Tmax and its thresholded mask with a threshold of more than 6 sec, mean transit time, cerebral blood flow, and cerebral blood volume maps. The number of channels is denoted on the top of the box. The skip connections allow detailed features to be maintained during training. In an attention gate, the output of previous layer (*g*) and the symmetric encoding layer (x^l) undergo convolution (with 1-by-1 kernel), summation, and ReLU activation. Then another convolution with sigmoid activation is applied to the extract attention coefficient (*a*), which is then multiplied with the skip connection.

©2020 Yu Y et al. *JAMA Network Open*.

eFigure 2. Example of Cases With Low, Medium, and High Dice Score Coefficient

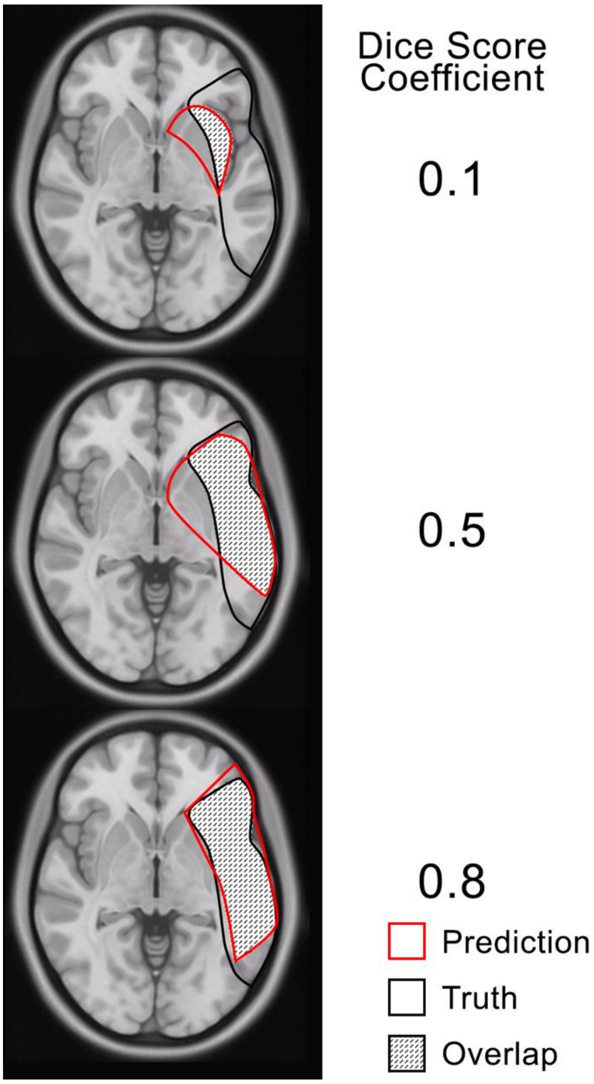

Three example cases with low, medium, and high Dice score coefficient (DSC) are shown. The more the overlap between the predicted lesion (red line) and the true lesion (black line) is, the higher the DSC will be. DSC of around 0.50 is usually considered significant overlap for this task. We used this metric to describe not only the accuracy of the prediction in terms of size, but also to make sure it detects the correct location as well.

©2020 Yu Y et al. *JAMA Network Open*.

eFigure 3. The Correlation of Cubic-Rooted Volume Prediction From Model vs True Lesion Volume

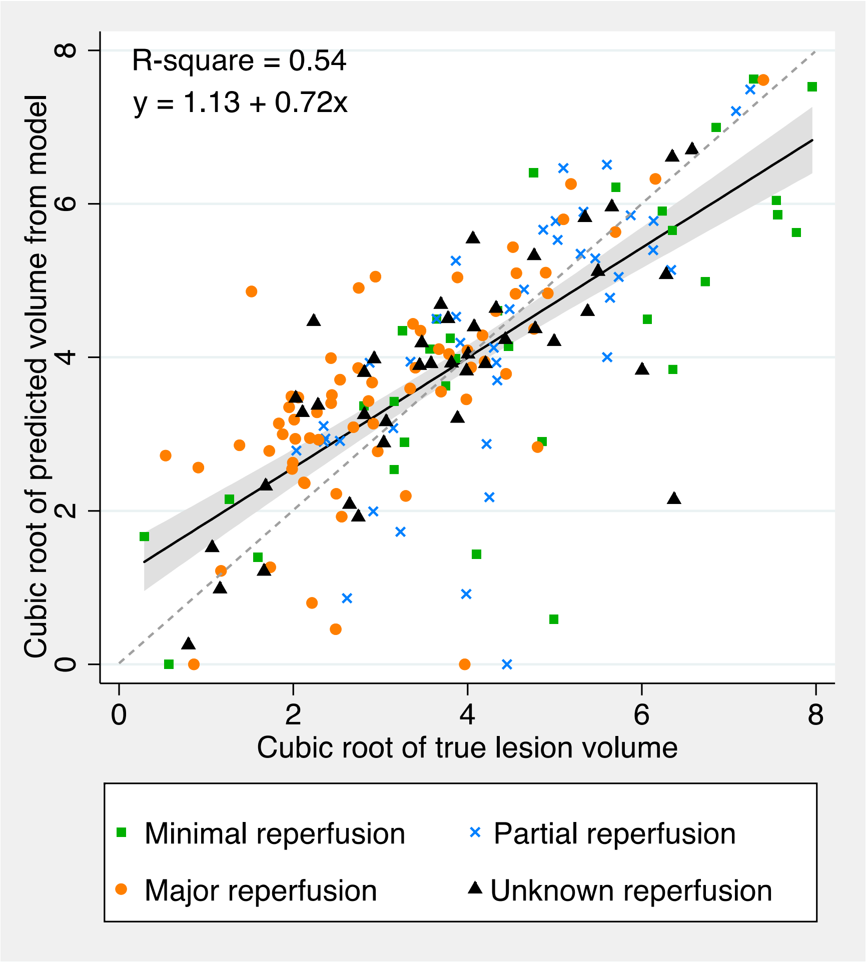

The black line represents the fitted linear function of all cases, and grey area represents 95% confidence interval.

©2020 Yu Y et al. *JAMA Network Open*.

eFigure 4. Comparison Between the Proposed Deep Learning Model, Tmax + ADC, and ADC Lesion Volume Prediction in Patients With Minimal, Partial, Major, and Unknown Reperfusion

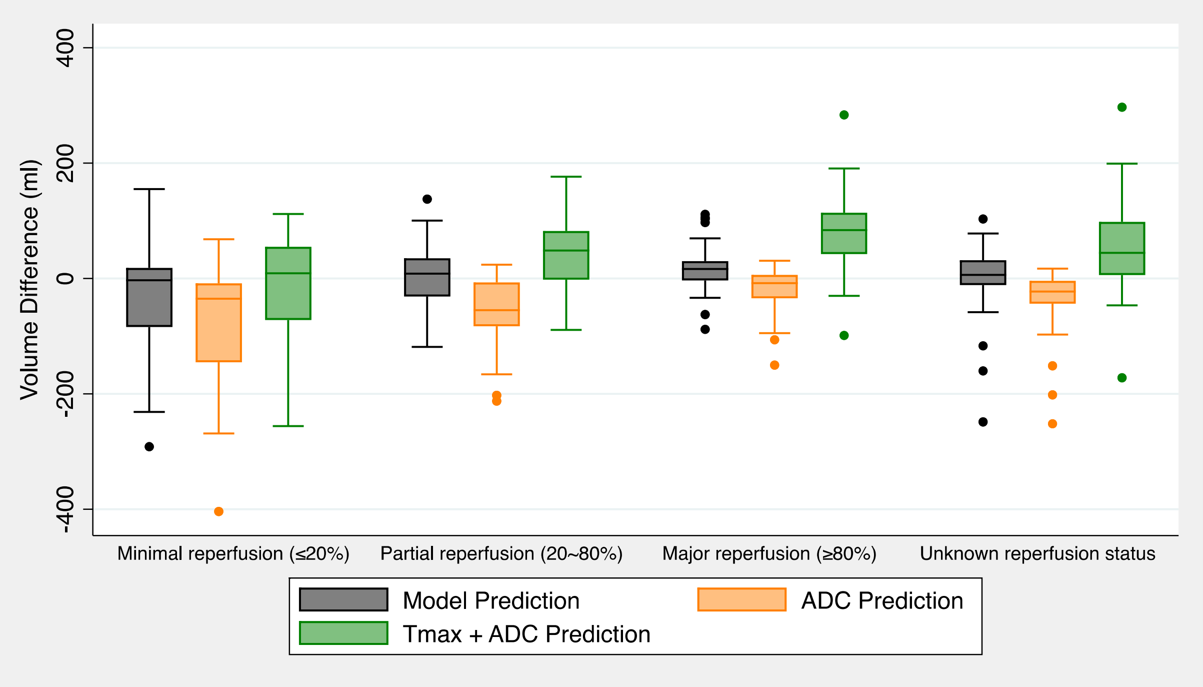

The deep learning model prediction is more stable across all subgroups with a mean volume difference closer to zero compared to the predictions of ADC and Tmax. The line inside the box represents median volume difference. The boundaries of boxes represent 25th and 75th percentile of volume difference. The error bar represents upper and lower 95% confidence intervals.

©2020 Yu Y et al. *JAMA Network Open*.

eFigure 5. Examples of Predictions From Model Compared With Thresholding Methods in Atypical Cases

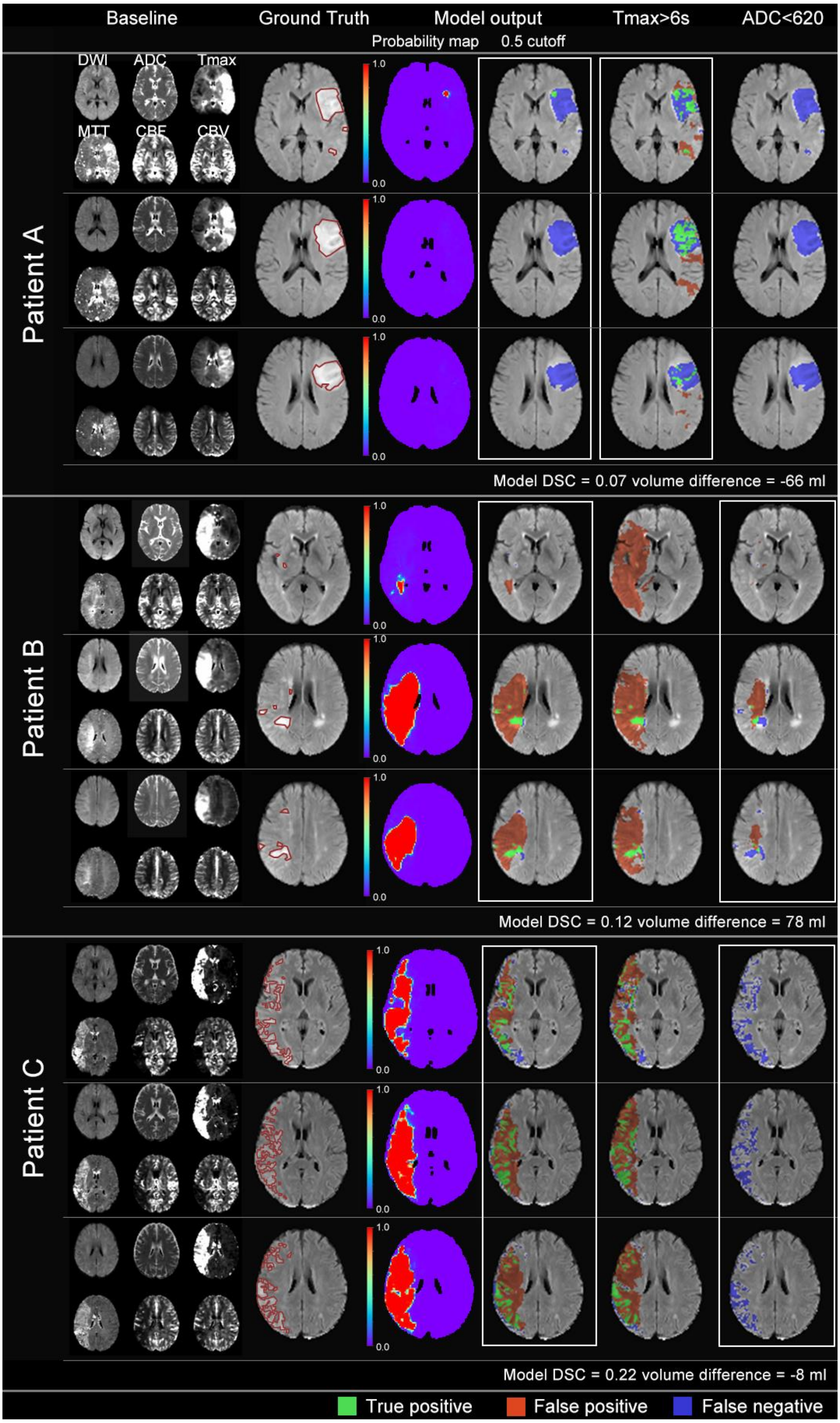

©2020 Yu Y et al. *JAMA Network Open*.

A) Initial negative DWI: A male patient with baseline NIHSS of 8 and a negative baseline DWI who received IV tPA and thrombectomy at 8.8 hrs after onset, achieved TICI 2b recanalization and 24 hr reperfusion rate of 15%. However, the patient still had a significant lesion growth and the model fails to predict the infarct lesion. In this case, the Tmax+ADC method performed best. B) DWI reversal: A female patient with baseline NIHSS of 20 who underwent thrombectomy at 4 hrs after onset with TICI 2b recanalization. In this case, the ADC thresholding method performed best, although it is possible that some of the regions with DWI reversal may represent infarcted tissue on later follow-up. C) A male patient with baseline NIHSS of 14, who underwent thrombectomy at 1.6 hrs and achieved TICI 3 recanalization and 24 hr reperfusion rate of 83%. Despite that, the patients still developed infarctions in cortical regions. The ADC model predicted no lesion, while the Tmax+ADC overpredicted the lesion by 114 ml. The proposed model predicted an intermediate sized lesion and had the best performance in this case.

Abbreviations: NIHSS = National Institute of Health Stroke Sclae, mRS = modified Rankin Scale, TICI = Thrombolysis in Cerebral Infarction, IV tPA = Intravenous tissue Plasminogen Activator, DWI = diffusion-weighted imaging.

©2020 Yu Y et al. *JAMA Network Open*.

eReferences.

1. Oktay O, Schlemper J, Le Folgoc L, et al. Attention U-Net: Learning Where to Look for the Pancreas. *arXiv e-prints.* 2018. https://ui.adsabs.harvard.edu/\#abs/2018arXiv180403999O. Accessed April 01, 2018.
2. Srivastava N, Hinton G, Krizhevsky A, Sutskever I, Salakhutdinov R. Dropout: A Simple Way to Prevent Neural Networks from Overfitting. *J Mach Learn Res.* 2014;15:1929-1958.
3. Nielsen A, Hansen MB, Tietze A, Mouridsen K. Prediction of Tissue Outcome and Assessment of Treatment Effect in Acute Ischemic Stroke Using Deep Learning. *Stroke; a journal of cerebral circulation.* 2018;49(6):1394-1401.
4. Jonsdottir KY, Ostergaard L, Mouridsen K. Predicting tissue outcome from acute stroke magnetic resonance imaging: improving model performance by optimal sampling of training data. *Stroke; a journal of cerebral circulation.* 2009;40(9):3006-3011.
5. McKinley R, Hani L, Gralla J, et al. Fully automated stroke tissue estimation using random forest classifiers (FASTER). *Journal of cerebral blood flow and metabolism : official journal of the International Society of Cerebral Blood Flow and Metabolism.* 2017;37(8):2728-2741.
6. Pinto A, McKinley R, Alves V, Wiest R, Silva CA, Reyes M. Stroke Lesion Outcome Prediction Based on MRI Imaging Combined With Clinical Information. *Front Neurol.* 2018;9:1060.
7. Stier N, Vincent N, Liebeskind D, Scalzo F. Deep Learning of Tissue Fate Features in Acute Ischemic Stroke. *Proceedings (IEEE Int Conf Bioinformatics Biomed).* 2015;2015:1316-1321.
8. Winzeck S, Hakim A, McKinley R, et al. ISLES 2016 and 2017-Benchmarking Ischemic Stroke Lesion Outcome Prediction Based on Multispectral MRI. *Front Neurol.* 2018;9:679.
